# Brazilians’ level of knowledge, attitudes and practices towards COVID-19: a cross-sectional study

**DOI:** 10.1590/1516-3180.2021.0541.23072021

**Published:** 2022-03-18

**Authors:** Rodrigo Galvão Bueno Gardona, José Vitor da Silva, Gisele Arruda, Silvana Damin, Edson Abdala, Christiana Almeida Salvador Lima, Leonardo de Souza Vasconcellos, Wladimir Queiroz, Alini Cristini Zandonái, Ailla Mazon Danielsk, Ana Carolina Villar de Sena, Álvaro Cesar Cattani, Amanda Bringhentti, Angélica Denardi, Ana Lígia Scotti Alérico, Gabriella Fergutz, Izabela de Oliveira Ribas, Laura Maria Voss Spricigo, Lara Gandolfo, Liamara Correa, Jardel Cristiano Bordignon, Juliana Girotto de Oliveira, Michel Pandolfo Stefanel, Beatriz Castro Reis, Vilson Geraldo de Campos, Danilo Ortigoso, Gerusa Maria Figueiredo

**Affiliations:** I MSc, PhD. Medical Undergraduate Student, Centro Universitário de Pato Branco (UNIDEP), Pato Branco (PR), Brazil.; II MSc, PhD. Professor, School of Nursing, Universidade Federal de Alfenas (UFAL), Alfenas (MG), Brazil.; III MSc, PhD. Professor, Department of Health Sciences, Universidade Estadual do Oeste do Paraná (UNIOESTE), Francisco Beltrão (PR), Brazil.; IV MSc, PhD. Scholarship Biologist, Department of Health Sciences, Universidade Estadual do Oeste do Paraná (UNIOESTE), Francisco Beltrão (PR), Brazil.; V MD, MSc, PhD. Professor, Department of Infectious and Parasitic Diseases, Faculdade de Medicina da Universidade de São Paulo (USP), São Paulo, Brazil.; VI MSc, PhD. Professor, Centro Universitário de Pato Branco (UNIDEP), Pato Branco (PR), Brazil.; VII MD, MSc, PhD. Professor, Medical School of Universidade Federal de Minas Gerais, Belo Horizonte (MG), Brazil.; VIII MD, MSc. Coordinator, Research Institutes, Instituto de Infectologia Emílio Ribas, São Paulo (SP), Brazil.; IX Medical Undergraduate Student, Centro Universitário de Pato Branco (UNIDEP), Pato Branco (PR), Brazil.; X Medical Undergraduate Student, Centro Universitário de Pato Branco (UNIDEP), Pato Branco (PR), Brazil.; XI Medical Undergraduate Student, Centro Universitário de Pato Branco (UNIDEP), Pato Branco (PR), Brazil.; XII MSc. Professor, Centro Universitário de Pato Branco (UNIDEP), Pato Branco (PR), Brazil.; XIII Medical Undergraduate Student, Centro Universitário de Pato Branco (UNIDEP), Pato Branco (PR), Brazil.; XIV Medical Undergraduate Student, Centro Universitário de Pato Branco (UNIDEP), Pato Branco (PR), Brazil.; XV Medical Undergraduate Student, Centro Universitário de Pato Branco (UNIDEP), Pato Branco (PR), Brazil.; XVI Medical Undergraduate Student, Centro Universitário de Pato Branco (UNIDEP), Pato Branco (PR), Brazil.; XVII Medical Undergraduate Student, Centro Universitário de Pato Branco (UNIDEP), Pato Branco (PR), Brazil.; XVIII Medical Undergraduate Student, Centro Universitário de Pato Branco (UNIDEP), Pato Branco (PR), Brazil.; XIX Medical Undergraduate Student, Centro Universitário de Pato Branco (UNIDEP), Pato Branco (PR), Brazil.; XX Medical Undergraduate Student, Centro Universitário de Pato Branco (UNIDEP), Pato Branco (PR), Brazil.; XXI MSc. Professor, Instituto Federal do Paraná (IFPR), Centro Universitário de Pato Branco (UNIDEP), Pato Branco (PR), Brazil.; XXII Medical Undergraduate Student, Centro Universitário de Pato Branco (UNIDEP), Pato Branco (PR), Brazil.; XXIII Medical Undergraduate Student, Centro Universitário de Pato Branco (UNIDEP), Pato Branco (PR), Brazil.; XXIV MD. Neonatologist, Hospital Regional do Sudoeste - Dr. Walter Alberto Pecoits (HRS), Francisco Beltrão (PR); and Professor, Centro Universitário de Pato Branco (UNIDEP), Pato Branco (PR), Brazil.; XXV MD. Professor, Centro Universitário de Pato Branco (UNIDEP), Pato Branco, PR, Brazil.; XXVI BA. Journalist, Self-employed journalism/medical press office, São Paulo (SP), Brazil.; XXVII MD, MSc, PhD. Professor, Department of Preventive Medicine and Institute of Tropical Medicine (IMT), Faculdade de Medicina da Universidade de São Paulo (USP), São Paulo (SP), Brazil.

**Keywords:** Behavior, Public health, COVID-19, SARS-CoV-2, Information, Conduct, 2019 novel coronavirus

## Abstract

**BACKGROUND:**

Brazil is facing increasing cycles of numbers of infected people and deaths resulting from coronavirus disease 2019 (COVID-19). This situation involves a series of factors, including the behavior of the population, that can be decisive for controlling the disease.

**OBJECTIVE:**

To determine the knowledge, attitudes and practices of the Brazilian population regarding COVID-19.

**DESIGN AND SETTING:**

Cross-sectional survey-type study, conducted using a population sample from different Brazilian states.

**METHODS:**

A quantitative, descriptive and analytical approach was used. Sampling was done according to convenience and via snowballing. The data collection instrument was a knowledge, attitudes and practices system.

**RESULTS:**

1,655 people from all over Brazil participated in the survey; 80% were living in the southern region and 70.15% were female. More than 90% had knowledge and good attitudes relating to the means of transmission, preventive care and symptoms associated with COVID-19, although their knowledge and attitudes were not fully reflected in daily practices, for which there was lower adherence (80%). Greater knowledge was correlated with older participants, larger number of children, female sex and marital status; better attitude, with female sex and complete higher education; and better practices, with greater age, larger number of children and female sex.

**CONCLUSION:**

A large part of the population has general knowledge about COVID-19, but not all knowledge was applied in practice. Older people, females and university graduates stood out as the best informed and most committed to controlling the disease.

## INTRODUCTION

Through the etiological agent severe acute respiratory syndrome coronavirus 2 (SARS-CoV-2), coronavirus disease 2019 (COVID-19) has become responsible for causing respiratory^1,2^ and systemic^3^ disorders among many thousands of people around the world, including severe expressions. These conditions are related to individuals’ intense and disordered multisystemic inflammatory response to the disease.^1,2^

COVID-19 has already infected more than 178,927,817 people and has led to the death of at least 3,875,915 people worldwide. In South America, about 500,000 people have lost their lives to this disease in Brazil alone. In the first half of 2021, Brazil was experiencing its worst time regarding the numbers of cases/deaths since the beginning of the pandemic.^4^

Therefore, despite the advances in vaccines against SARS-CoV-2, the classic methods of non-pharmacological prevention and control, such as proper hand hygiene with alcohol gel and use of masks, need to be continued and encouraged,^5^ along with social distancing, especially for high-risk groups.^6^ Studies have confirmed that the speed with which new cases of COVID-19 occur reduces as social isolation increases.^7^

It is likely that adherence to non-pharmacological prevention measures and the success or failure of such actions are closely influenced by the population’s awareness of the subject,^8^ which is endorsed by the theory of knowledge, attitudes and practices. This theory, which was first put forward in the 1960s, assumes that suitable health-related behavior is divided into three continual processes, “the acquisition of knowledge, the generation of attitudes and the change in behavior itself”.^9^ From this perspective, knowledge is the determining factor for a change in behavior, while beliefs and attitudes are the “driving force” of this change.^9,10^ This theory builds on the foundations of another concept that was created in 1950, “the health belief model”, which argues that belief is essential for people to adopt healthy behavior, based on preventive pillars.^11^

Through the media and official health agencies, information about COVID-19 has been intensively disseminated. However, given the continuing debate and scientific advances relating to preventive care, diagnostic criteria and treatment, this knowledge does not remain static.^12^ Nonetheless, it needs to be asked whether there is any process of acquisition of knowledge and adherence to attitudes/practices regarding preventive measures against COVID-19 among people in Brazil.

Understanding these attitudes, or lack of them, during the pandemic can show up any bottlenecks that may exist and the reasons for failure, in situations relating to the great present challenge of ensuring the safety and effective protection of the population.^13^

## OBJECTIVE

Thus, the aim of this study was to assess the level of knowledge, attitudes and practices of the Brazilian population towards COVID-19, in relation to its sociodemographic characteristics, through using a method for ascertaining knowledge, attitudes and practices.

## METHODS

The present study received prior approval from the National Research Ethics Committee (Comissão Nacional de Ética em Pesquisa, CONEP) on April 3, 2020, under the number 3.982.636, as provided for in Resolution 466/2012 of the National Health Council. The research that was developed was of survey type, with a quantitative descriptive-analytical approach. The sampling used was obtained according to convenience and through snowballing, given the possible ignorance of the study participants. This method enabled identification and integration of the sample through third-party references.^14^

We spread word of this study among our existing contacts through the instant messaging application WhatsApp. Participants thus recruited were asked to send invitations randomly to their telephone contacts, to try to also enroll them as participants in this research. We aimed to reach the largest number of people and different audiences possible, and to enable participation and responses from all Brazilian states.

The messages sent out provided a link to access an electronic form that we developed on Google Forms. This containing the informed consent statement, guidelines for resolution, and study questions to be answered by people aged 18 years and over. For people who agreed to participate in the study, but who because of limitations indicated that they were unable to provide responses through the form (this was especially the case among elderly participants), the form was applied via telephone, in accordance with the ethical guidance of Resolution No. 510/2016 of the National Health Council.^15^

Data collection took place from July to December 2020. The sample size was calculated using a scale based on the items/subject ratio and at least five participants per item, as proposed by Pasquali.^16^ In this study, approximately 71 participants were used per item, with the highest possible number of members. This was a decisive factor with regard to the internal consistency of the scale.^16^

Individuals from 23 Brazilian states took part in the study. The only states from which there were no responses were Roraima, Amapá and Amazonas. Participants answered a questionnaire containing 23 questions with three choices: a) true; b) false; and c) I don’t know. These questions sought to recognize clinical and epidemiological knowledge;^15^ attitudes and practices in relation to COVID-19;^17^ and sociodemographic characteristics (age, sex, marital status, education, state of origin, profession and children).^18^ This was adapted from a previously published knowledge, attitudes and practices system.^9^

The questions in this instrument addressed information and concepts set forth by the World Health Organization,^18^ regarding etiological agents, transmission routes, protection routes, signs and symptoms, the most vulnerable populations, care and related beliefs. The instrument was built for use in a general population in which people might be unfamiliar with the subject. Therefore, easily understood questions were recommended: these were evaluated from the semantic, conceptual and cultural points of view by three consultants, who were specialists in the field of infectious diseases and in the methodology of this study.

The responses to all the questions were organized to enable association analysis and were counted in a scoring system. Each item in the knowledge, attitudes and practices system consisted of a statement with three alternatives: a) true; b) false; and c) I don’t know. The alternatives “false” and “I don’t know” were subsequently condensed into a single “false” alternative^19^ because of the small number of statements of the type “I don’t know”.

The assertions were graded as correct or incorrect, and scores were calculated from the sum of these responses. Continuous/numerical variables were then subjected to descriptive analysis (mean, median and standard deviation); and categorical/qualitative variables were subjected to absolute and relative frequency analysis.

Regarding the scores per domain, the maximum score for knowledge was 13, while for practices and attitudes it was five points. Initially, the data were subjected to a normality test (Kolmogorov-Smirnov). Because the data were found not to adhere to normal distribution, nonparametric tests were used for quantitative numerical correlations (age and number of children) and Spearman’s correlation coefficient was calculated. For comparative analysis on nominal qualitative variables, the Mann-Whitney test was used for sex and the Kruskal-Wallis test with Tukey’s multiple comparisons was used for marital status and educational level. Variables for which the correlations were significant (P < 0.05) were integrated with the multiple regression analysis for each score, which was performed by using the stepwise method, in the SPSS for Windows software, version 19.0.0 (SPSS, São Paulo, Brazil).

## RESULTS

One thousand six hundred and fifty-five people participated in the survey, of whom 1,161 (70.15%) were women, and 494 (29.84%) were men. The participants were aged between 18 and 92, with an average age of 35 (SD 14.55). Regarding marital status, 804 (48.55%) of the participants reported being married or in a stable relationship, and 754 (45.53%) were single. Approximately 50% had children. The predominant level of education among the participants was complete higher education (54.65%), followed by incomplete higher education (28.44%). The majority had some form of occupation at the time when they answered the questionnaire. Among the participants, 650 (39.27%) said that they had a job in companies, 226 (13.65%) worked in education and 189 (11.41%) were students. The other 590 individuals (35.67%) were distributed in other less frequent functions (Table 1).

**Table 1.** Sociodemographic characteristics of the study participants

	Variables	Frequency (n)	%
**Sex**	Female	1161	70.15
	Male	494	29.85
**Age (years)**	18-37	947	57.22
	38-57	553	33.41
	58-77	148	8.94
	78-92	7	0.42
**Marital status**	Single	754	45.53
	Married/stable union	804	48.55
	Widowed	16	0.97
	Divorced	81	4.89
**Children**	Yes	808	48.79
	No	847	51.15
**Schooling**	Incomplete elementary school	22	1.33
	Complete elementary school	18	1.09
	Incomplete high school	15	0.91
	Complete high school	224	13.53
	Incomplete higher education	471	28.44
	Complete higher education	905	54.65
**Home state**	Acre	2	0.12
	Alagoas	1	0.06
	Bahia	15	0.90
	Ceará	4	0.24
	Distrito Federal	2	0.12
	Espírito Santo	5	0.30
	Goiás	3	0.18
	Maranhão	5	0.30
	Mato Grosso	23	1.38
	Mato Grosso do Sul	16	0.96
	Minas Gerais	48	2.90
	Pará	4	0.24
	Paraíba	1	0.06
	Paraná	1041	62.90
	Pernambuco	7	0.42
	Piauí	4	0.24
	Rio de Janeiro	44	2.65
	Rio Grande do Norte	5	0.30
	Rio Grande do Sul	124	7.49
	Rondônia	9	0.54
	Santa Catarina	169	10.20
	São Paulo	121	7.31
	Sergipe	1	0.06
	Did not answer	1	0.06
**Profession/Occupation**	Company employee	650	39.27
	Educational institution employee	226	13.65
	Distribution company employee	91	5.49
	Financial services company employee	35	2.11
	Student	189	11.41
	Retiree	74	4.47
	Unemployed	22	1.32
	Domestic employee	43	2.59
	Communication service employee	34	2.05
	Construction company employee	17	1.02
	Environmental services company employee	4	0.24
	Healthcare institution employee	45	2.71
	Tourist services employee	12	0.72
	Entertainment company employee	18	1.08
	Transportation company employee	6	0.36
	Other types of employment	59	3.56
	Did not answer	130	7.85

Among the interviewees, 1,334 (80%) were from the southern region, 218 (13.18%) from the southeast, 44 (2.66%) from the center-west, 43 (2.59%) from the north and 15 (0.90%) from the northeast. The participation of 1,041 individuals (62.90%) living in the state of Paraná can be highlighted: this was the state in which the distribution of the questionnaire began (Table 1).

In the system for evaluating the knowledge, attitudes and practices of the participants in relation to COVID-19, the highest score (percentage of correct answers) was in the knowledge domain (94.84%), followed by the score in the attitudes domain (92.20%). The lowest performance, i.e. the lowest number of correct responses from the participants (80.00%), was in relation to the questions in the practical domain (Table 2).

**Table 2.** Frequency of correct responses, overall mean and percentage mean (%) of statements relating to the knowledge, attitudes and practices of the study participants towards coronavirus disease 2019 (COVID-19)

Statements relating to the knowledge domain	Frequency of correct responses (n = 1,655)
01. Does COVID-19 cause respiratory problems?	1,507 (91.06%)
02. Are fever and coughing common?	1,400 (84.59%)
03. Are a runny nose and sneezing less common?	894 (54.02%)
04. Is it true that not everyone infected will progress to serious complications?	1,586 (95.83%)
05. Are younger people, elderly people and immunosuppressed children more vulnerable?	1,447 (87.43%)
06. Does transmission occur by means of droplets?	1,618 (97.76%)
07. Is social isolation effective for disease prevention?	1,625 (98.19%)
08. Should I avoid crowded places?	1,630 (98.49%)
09. Doesn’t the flu vaccine prevent COVID 19?	1,608 (97.16%)
10. Should people with flu symptoms undergo isolation?	1,513 (91.42%)
11. Should I seek a healthcare service if I have fever and respiratory distress?	1,631 (98.55%)
12. Can I use soap and water instead of alcohol gel (community level)?	1,497 (90.45%)
13. Should I wash my hands with soap and water for 20 seconds (rubbing my hands together)?	1,583 (96.65%)
Overall mean number of correct responses	11.81 ± 1.18 (94.84%)
**Statements relating to the attitudes domain**	**Frequency of correct responses** **(n = 1,655)**
01. WHO and Ministry of Health recommendations are effective.	1,555 (93.96%)
02. Social isolation reduces contamination.	1,605 (96.98%)
03. I don’t follow the guidelines because I believe the disease is not that serious.	1,545 (93.35%)
04. I am concerned about the damage caused by the disease (health, unemployment and other matters).	1,604 (96.92%)
05. I believe the pandemic is temporary, despite the difficulties.	1,318 (79.64%)
Overall mean number of correct responses	4.61 ± 0.67 (92.20%)
**Statements relating to the practices domain**	**Frequency of correct responses** **(n = 1,655)**
01. I’ve been in public places in the last few days.	800 (48.34%)
02. I go to public places even when living with a high-risk group.	251 (15.17%)
03. I carry out hand washing and social distancing.	1,600 (96.68%)
04. I carry out cleaning of belongings and objects.	1,218 (73.60%)
05. I greet people with gestures.	1,550 (93.66%)
Overall mean number of correct responses	4.00 ± 0.97 (80.00%)

In the knowledge domain, about 98% of the participants showed that they knew that “COVID-19 has droplet transmission”, that “the flu vaccine does not prevent COVID-19”, that “they should avoid crowded places” and that “social isolation is effective for prevention of COVID-19.” Moreover, about 88% recognized that “younger people, elderly people and immunosuppressed children form a group that is more vulnerable to the disease”. When asked about preventive care, such as “rubbing one’s hands together for 20 seconds” during “hand washing with soap and water, and use of alcohol gel”, the average percentage of correct responses (knowledge among the participants) also decreased to 96.60% and 90.40%, respectively (Table 2).

It was found that 98.50% of the participants were aware that they “should seek the healthcare service in the event of fever and respiratory distress”, but on the contrary, almost 10% of them did not recognize that “COVID-19 can cause respiratory problems”, and another 15% did not recognize that “fever and cough are common in COVID-19”. In addition, almost half of the participants (46%) were unaware that symptoms such as “runny nose and sneezing are less common” among individuals infected with this disease (Table 2).

Regarding attitudes, approximately 6% of the study participants did not “believe in the effectiveness of the World Health Organization and Ministry of Health recommendations” and did not “follow them”. For some of them (3.02%), this was because they considered that “social isolation does not decrease contamination”; while for others (6.65%), this was because they did not believe in “the severity of the disease.” However, a higher percentage of participants (20%) considered that “the pandemic is non-transitory”, and 3.08% showed concern about the disease, through the belief that, directly or indirectly, “it is reflected in damage to health and employment” (Table 2).

Regarding the domain of the participants’ practices, almost 50% reported “having normally frequented public places”, even though 15.17% of them were “living with people in the high-risk group”. Although a large part of the population continued to attend public places, the majority (96.68%) claimed to carry out “hand washing” and “social distancing” in public places and had started to “greet people with gestures” (93.66%). However, in their routine, “sanitizing your belongings and objects” was not a practice for 26.40% of the participants.

Correlations between sociodemographic variables and the knowledge, attitudes and practices system showed differences with regard to knowledge, attitudes and practices (Tables 3, 4 and 5).

**Table 3.** Average knowledge score versus demographic variables (age, number of children, sex, marital status and educational level of the participants)

Variables	Knowledge score		
**Age**	Correlation coefficient	(P)	Total (n)
	0.173	< 0.001	1,655
**Number of children**	Correlation coefficient	(P)	Total
	0.156	< 0.001	1,655
**Sex**	Median (mean ± SD)	(P)	Total (n)
Male	12.00 (11.59 ± 1.25)		494
Female	12.00 (11.90 ± 1.14)	< 0.001	1,161
**Marital status**	Median (mean ± SD)	(P)	Total (n)
Single	12.00 (11.62 ± 1.18)		754
Married	12.00 (11.98 ± 1.13)	< 0.001	670
Widowed	12.00 (12.31 ± 0.60)		16
Divorced	12.00 (11.80 ± 1.54)		81
Stable union	12.00 (11.91 ± 1.04)		134
**Educational level**	Median (mean ± SD)	(P)	Total (n)
Incomplete elementary school (1)	11.00 (12.00 ± 2.45)	1 < 6	22
Complete elementary school (2)	11.67 (12.00 ± 1.24)		18
Incomplete high school (3)	11.53 (12.00 ± 1.64)		15
Complete high school (4)	11.66 (12.00 ± 1.40)	4 < 6	224
Incomplete higher education (5)	11.63 (12.00 ± 1.16)	5 < 6	471
Complete higher education (6)	11.96 (12.00 ± 1.05)	< 0.001	905

Note: To correlate knowledge, sex and marital status, the Mann-Whitney and Kruskal-Wallis tests were used, respectively. SD = standard deviation.

**Table 4.** Average score for attitudes versus demographic variables (sex and educational level of the participants)

Variables	Attitude score		
**Sex**	Median (mean ± SD)	(P)	Total (n)
Male	5.00 (4.54 ± 0.75)		494
Female	5.00 (4.64 ± 0.63)	0.034	1,161
**Educational level**	Median (mean ± SD)	(P)	Total (n)
Incomplete elementary school	4.00 (4.00 ± 1.23)		22
Complete elementary school	5.00 (4.44 ± 0.70)		18
Incomplete high school	4.00 (4.07 ± 0.70)		15
Complete high school	5.00 (4.56 ± 0.73)		224
Incomplete higher education	5.00 (4.57 ± 0.70)		471
Complete higher education	5.00 (4.67 ± 0.61)	0.038	905

Note: For multiple comparison of attitudes versus the educational level variable, Tukey’s multiple comparison test was used. SD = standard deviation.

**Table 5.** Average score for practices versus demographic variables (age, number of children and sex of the participants)

Variable	Practices score		
**Age**	Correlation coefficient	(P)	Total (n)
	0.061	0.014	1,655
**Number of children**	Correlation coefficient	(P)	Total (n)
	0.054	0.028	1,655
**Sex**	Median (mean ± SD)	(P)	Total (n)
Male	4.00 (3.77 ± 1.01)		494
Female	4.00 (4.09 ± 0.93)	< 0.001	1,161

Note: To correlate practices with the variable of sex, the Mann-Whitney test was used. SD = standard deviation.

The variables of age, number of children, sex, marital status and education were correlated with the knowledge domain (P = 0.001). Greater age, larger number of children and longer education correlated with greater knowledge. Females and married people had higher scores for this domain than males and single people (Table 3).

Regarding the attitudes domain, it was noted that females had higher scores (P = 0.034). There was also an association between education and the attitudes of the participants. The categories of complete high school and incomplete higher education did not differ from each other; however, they presented a lower score than complete higher education (Table 4).

Regarding practices, there was a correlation between age and the number of children of the participants. Older age and a larger number of children increased adherence to correct practices (P = 0.014 for age; and P = 0.028 for the number of children). As in the other domains, females also had a high mean score than males (P = 0.001) (Table 5).

Univariate analyses in the multiple regression model also showed differences within the knowledge, attitudes and practices system in relation to demographic variables (Table 6). Females and individuals with higher education had a better relationship with the knowledge domain (Beta = 0.302 for sex; and Beta = 0.233 for education) and also with the attitudes domain (Beta = 0.100 for sex; and Beta = 0.128 for education). Furthermore, female sex also had a greater association with practice scores (Beta = 0.134).

**Table 6.** Multiple regression analysis: knowledge, attitudes and practices in the study population

Knowledge, attitudes and practices system		Non-standardized coefficients		Standardized coefficients		
		Beta	Standard error	Beta	t	Sig*
**Knowledge**	Constant		0.074		156.673	0.000
	Complete higher education	0.233	0.062	0.098	3.784	0.000
	Female	0.302	0.062	0.117	4.868	0.000
	Single	-0.256	0.061	-0.108	-4.183	0.000
	Incomplete elementary school	-0.731	0.250	-0.071	-2.922	0.004
**Attitudes**	Constant	4.539	0.024		185.822	0.000
	Complete higher education	0.128	0.033	0.095	3.864	0.000
	Constant	4.468	0.035		127.459	0.000
	Complete higher education	0.128	0.033	0.095	3.878	0.000
	Female	0.100	0.036	0.068	2.792	0.005
**Practices**	Constant	3.385	0.030		113.540	0.000
	Single	-0.242	0.044	-0.133	-5.476	0.000
	Constant	3.347	0.033		102.262	0.000
	Single	-0.246	0.044	-0.136	-5.580	0.000
	Female	0.134	0.048	0.068	2.787	0.005

*Sig = significant.

## DISCUSSION

The overall results from this study revealed issues relating to knowledge, attitudes and practices regarding COVID-19 in Brazil. These have been undergoing extensive discussion and continual updating through the media, government bodies and the scientific community since the beginning of the pandemic.

Importantly, the method for evaluating knowledge, attitudes and practices has been adapted, built and used in other studies in different countries, depending on the cultural context and local reality.^8,20,21^ As shown in Table 1, most of the population assessed had completed higher education (54.65%), similar to what was shown in other studies.^20–22^

The high scores observed for the knowledge domain generally demonstrated that the Brazilian population has knowledge about COVID-19, especially regarding the means of transmission of SARS-CoV-2 and the care required for avoiding this (96%).

Knowledge about COVID-19 in different parts of the world is quite high, but it varies depending on the region.^23^ Surveys from two countries on different continents illustrate the differences in subjects’ levels of knowledge: the levels ranged from 62% in Paraguay^20^ to 90% in China.^21^ The latter percentage was similar to what was found in the present study (94.84%) (Table 2).

The level of knowledge among participants in the present survey showed a relationship with their level of education. Individuals who had completed higher education had higher knowledge scores than those with incomplete elementary school, complete high school and incomplete higher education (Table 3).

On the other hand, in Nepal, for example, where 45.50% of the people had not had higher education, the average knowledge score was 60.00%.^24^

Regarding preventive measures, which were evaluated in relation to part of the population (10.00%), this knowledge was found to be limited. For example, there is misinformation about the use of soap and water as an efficient alternative to use of alcohol gel for hand hygiene, or in the case of absence of alcohol gel. Similarly, in India, the majority of “educated” people and healthcare professionals were aware of how infection occurs and what the preventive measures are. Even so, about 57.00% of the people did not recognize the disease as highly contagious, and almost 10.00% did not realize the importance of hand hygiene and social isolation.^25^

Along the same lines, a study carried out in the United States showed that the participants had good knowledge about the forms of transmission and symptoms. However, these individuals also showed some misconceptions: for example only 37.80% believed that use of an ordinary surgical mask was highly effective for preventing from COVID-19, and 25.60% thought that it was wise to avoid Chinese restaurants.^26^ In Pakistan, 54.70% of the subjects reported not knowing that physical contact was the main means for spreading the infection.^27^ Contrary to this, in the present study, 97.76% of the participants knew that droplets are the main means of infection transmission.

Appropriation of knowledge relating to preventive care and its updates among the entire population has become essential for controlling COVID-19. Understanding of simple measures that can be applied to indoor environments in situations of limited resources, which is the reality for many Brazilian households, can be highlighted.

Regarding symptoms, 45.98% of the participants in the present study mistakenly said that a runny nose and sneezing were common in COVID-19 (Table 3). However, these do not correspond to the classic signs and symptoms of the disease,^28^ which are mainly fever (around 90%), dry cough (67.7%-86%), fatigue (38.1%) and dyspnea (18.6%-80%).^18^ In the Philippines, a considerable proportion (89.5%) of the sample was able to point out coughing and sneezing as transmission routes.^23^

In Brazil, 15% of the participants did not correlate some of these symptoms with COVID-19. However, recognition of typical signs and symptoms of the disease and the degree of concern that these represent, given the clinical condition manifested, directs possibly infected people to seek medical and/or hospital care at the proper time.

Regarding attitudes, the mean score identified in this study was high, thus confirming the findings from recent studies conducted elsewhere.^23^ In this regard, although 20% of the participants noticed that the COVID-19 pandemic was not a temporary phenomenon, 7% still refused to accept and/or follow instructions from official bodies such as the World Health Organization and the Ministry of Health (mask use, hand-cleaning with 70% alcohol or soap and water, avoidance of personal contact and avoidance of situations of crowding, among others).^17^

We observed that attitudes were not necessarily reflected in practice, considering that 26% of the participants reported that they did not clean their belongings and objects, 50% continued to go to public places and 15% were living with high-risk people. Thus, although people may believe the recommendations of official bodies for controlling the COVID-19 pandemic, in practice these recommendations are not put into effect. A large part of the population continues to have contact with many people in their daily routine, perhaps because of work needs or the mistaken feeling that only people with comorbidities (whether respiratory, cardiac or multifactorial) are likely to have a worsened prognosis when affected by COVID-19.^29^ However, the real reason why people were going to public places was not investigated, which thus generates a limitation to our study. Nonetheless, it can be assumed that people were doing this in relation to work needs, as shown in a study carried out in Pakistan, where 59.3% of the subjects continued to attend mosques to pray, amid the pandemic.^27^

From correlations and multiple regression analyses, females stood out as having the highest scores in all domains, like in other recent studies that observed higher mean scores for knowledge among women.^20,22,30^ Different studies on infectious diseases have also show that females had greater knowledge of the subject than males.^31,32^

With increasing age and larger numbers of children, higher knowledge scores were observed among our Brazilian population. This was put into practice by this population, in combating the COVID-19 pandemic. These results corroborate the findings of most previous studies that investigated these socioeconomic characteristics.^20,22,23,33,34^ On the other hand, in a study carried out in central Nepal, younger people showed more substantial knowledge, according to the authors.^24^

People with higher education or who were undergoing training tended to have scattered knowledge, as demonstrated by the present study. There were correlations with the level of education, both for knowledge (Table 3) and for attitudes (Table 4). This was confirmed through regression analyses (Table 6), such that people with higher education had more adequate knowledge and attitudes towards COVID-19. Lower levels of education can be considered to be a risk factor for the spread of viral infectious diseases and for disease progression to death.^35^

It is important to highlight that the average score for practices was lower (80.00%) than the scores for knowledge and attitudes (94.84 and 92.20%). In the light of this difference, it can be inferred that knowledge alone does not guarantee good practices, or their maintenance. As the pandemic advances, non-compliance with non-pharmacological practices to protect against SARS-CoV-2 infection may become more common.

It is worth remembering that the data in this study were collected in 2020, at a time when respondents were mostly aligned with preventive practices. However, it can be seen in everyday life that many of the measures that were adopted in the middle of the pandemic are not implemented with the same rigor today. Thus, within the Brazilian scenario of restrictions, psychological disturbances (stress and depression)^1,36^ and narrative wars between the various levels of government, the politicization of information may have affected not only knowledge, but also especially the beliefs of people in this country. These beliefs are reflected in their attitudes and practices, as described in the published knowledge, attitudes and practices system.^9,10^ In addition, progressive weakening of preventive practices may already be a reality in 2021. This is very worrying and is one of the factors responsible for the successive waves of COVID-19.

Furthermore, limitations of the study relating to sample selection may have generated some sampling bias, given that the sample was concentrated in the southern region of the country and among people with higher education levels. Thus, based on this initiative, future applications of the knowledge, attitudes and practices system are suggested, in order to understand the population, considering the changes that are occurring with regard to the COVID-19 pandemic.

## CONCLUSION

Through using this knowledge, attitudes and practices system, it was found in this Brazilian population that there was a high level of knowledge about COVID-19. However, there was less commitment to practical application of this knowledge.

Furthermore, some well-informed and active social groups, like older women with children and individuals with higher education levels were noted. These groups showed greater implementation of actions to combat COVID-19. This is an important finding that should be directed towards COVID-19 coping actions for less active groups.
